# Effects of Concentration and Spin Speed on the Optical and Electrical Properties of Silver Nanowire Transparent Electrodes

**DOI:** 10.3390/ma14092219

**Published:** 2021-04-26

**Authors:** Xiaopeng Li, Jiayue Zhou, Dejun Yan, Yong Peng, Yong Wang, Qi Zhou, Kehong Wang

**Affiliations:** 1 College of Materials Science and Technology, Nanjing University of Science and Technology, Nanjing 210014, China; lxp11s009184@163.com (J.Z.); cheezhou@163.com (Q.Z.); wkh1602@126.com (K.W.); 2 China State Shipbuilding Corporation Huangpu Wenchong Shipbuilding Company Limited, Guangzhou 510715, China; 13B909097@hit.edu.cn (D.Y.); zhangdeku@sohu.com (Y.W.)

**Keywords:** silver nanowires, concentration, spin speed, sheet resistance, transmittance, *FoM*, *haze value*

## Abstract

In this paper, silver nanowires (AgNWs) with a diameter of 40 nm and a length of 45 μm were dispersed into an ethanol solution to prepare AgNW solutions with concentrations of 1, 2, and 3 mg/mL, respectively. The AgNW solutions were then deposited on a glass substrate using spin-coating at 1000, 2000, and 3000 rpm for 45 s, respectively, to prepare transparent electrodes. The results showed that the distribution of AgNWs on the substrate increased in density with the increase in the AgNW solution concentration and the decrease in spin speed. The effect of concentration on the distribution of AgNWs was greater than that of the spin speed. The transmittance of each electrode was between 84.19% and 88.12% at 550 nm, the average sheet resistance was between 20.09 and 358.11 Ω/sq, the highest *figure of merit* (*FoM*) was 104.42, and the lowest *haze value* was 1.48%. The electrode prepared at 1000 rpm with a concentration of 2 mg/mL and that prepared at 3000 rpm with a concentration of 3 mg/mL were very similar in terms of the average sheet resistance, transmittance at 550 nm, *FoM*, and *haze value*; thus, these two electrodes could be considered equivalent. The *haze value* of the electrode was positively correlated with the spin speed at low concentration, but that relationship became inverse as the concentration rose. For the AgNWs used in this experiment with an aspect ratio of 1125, the concentration of the AgNW solution should reach at least 2 mg/mL to ensure that the *FoM* of the electrode is greater than 35.

## 1. Introduction

As technology has been developed, the demand for new high-performance electronic devices has increased. New flexible electronic devices, such as flexible wearable devices, electronic skin, touch screens, and flexible solar cells, have attracted an upsurge in research due to their good folding and tensile properties. As an important part of new flexible electronic devices, flexible transparent electrodes have been widely studied nationally and internationally [1,2,3,4,5,6].

Flexible transparent electrodes not only need to have a high electrical conductivity and high transmittance, but also need to have a certain flexibility. Currently, the most widely used transparent electrode material is indium tin oxide (ITO) [7]. Although ITO has excellent transmittance and electrical conductivities, its poor mechanical flexibility and high cost make it unsuitable for the new generation of flexible electronic devices [8,9]. For this reason, scholars have successively proposed a variety of ITO alternatives, such as carbon nanotubes, graphene, conductive polymers, and metal nanostructures. Cao applied arrays of single-walled carbon nanotubes to high-performance electronics [10]. Ho invented stretchable and multimodal all-graphene electronic skin [11]. Kim fabricated highly efficient flexible organic light-emitting devices based on poly(3,4-ethylenedioxythiophene):poly(styrene sulfonate)(PEDOT:PSS) electrodes doped with highly conductive Pyronin B [12]. Kang reported the use of a silver nanowire network for triboelectric nanogenerators [13]. Wang mixed silver nanowires (AgNWs) and nanosilver-coated copper micronflakes (mass ratio 1:9) to prepare electrically conductive adhesives [14].

Among these alternatives, silver nanowires (AgNWs) are regarded as the ITO substitutes with the highest potential due to their excellent electrical conductivity, transmittance, and flexibility [15]. There are many synthetic methods for AgNWs, including the solution chemical reduction method and template method. Sun used ethylene glycol to restore AgNO_3_ to Ag nanoparticles at 160 °C, finally obtaining AgNWs with an aspect ratio of up to 1000 [16]. Cui synthesized AgNWs with a diameter of 50 nm and a length of 6 μm on a DNA template using an electrochemical technique [17]. The synthesized AgNWs are generally dispersed in an ethanol solution or isopropanol solution for preservation to avoid oxidation and vulcanization. 

The preparation methods of the transparent electrodes mainly include the spin-coating, spray-coating, drop-coating, rod-coating, brush-painting, and patterning methods. Wu studied the conductivity enhancement of PEDOT:PSS electrodes via the addition of chloroplatinic acid and its mechanism utilizing the spin-coating method [18]. Choi used a continuous two-step spray-coating method to prepare annealing-free, flexible AgNW-polymer composite electrodes [19]. Cui optimized the ethylene-glycol-doped PEDOT:PSS transparent electrodes by the drop-coating method [20]. Huang chose a Mayer rod-coating method to prepare AgNW electrodes [21]. Lee proposed the preparation of transparent PEDOT/AgNWs/PEDOT multilayer electrodes by brush painting [22]. Ko invented a simple AgNW patterning method based on poly(ethylene glycol) photolithography [23].

The laboratory usually adopts the convenient spin-coating method. First, the wet film of AgNWs was prepared by a spin coater, and after the solvent volatilized, the dry AgNW transparent electrode could be obtained. The optical and electrical properties of the spin-coated AgNW transparent electrode are mainly affected by two factors. One is the specification of AgNWs, such as the aspect ratio, concentration, viscosity, and dispersion in the solvent, which can be regulated by adjusting the synthesis method of AgNWs and the subsequent purification process. The other is the spin process, which can be regulated by adjusting the spin speed and the duration.

In recent years, scholars have successfully applied AgNW transparent electrodes to a variety of electronic devices. By embedding AgNWs into polymethyl methacrylate (PMMA), Kim prepared a flexible transparent electrode with a low roughness and successfully applied it to a flexible organic light-emitting diode (OLED) device, where the on–off voltage of the OLED device was 2.8 V and its maximum brightness reached 23,741 cd/m^2^ [24]. Seo applied AgNW transparent electrodes to a flexible solar cell, whose short-circuit current density, open-circuit voltage, filling factor, series resistance, and energy conversion efficiency were, respectively, 17.4 mA/cm^2^, 0.764 V, 64.2%, 4.13 Ω/cm^2^, and 8.75%, showing a good mechanical flexibility [25]. Li treated the AgNW transparent electrode with plasma and applied it to an OLED device. The maximum brightness of the OLED device reached 27,000 cd/m^2^ and its current efficiency was 11.8 cd/A [26]. The research carried out by Norio showed that stable electrical insulation with a small removal trace area of AgNWs can be expected by using a proper long-pulse duration in nanosecond pulsed laser processing, which provided a new idea for the application of AgNW transparent electrodes in devices [27].

At present, AgNW transparent electrodes have been widely used in various electronic devices; however, as the electrode is only one of the components of electronic devices, scholars have paid more attention to the overall performance parameters of electronic devices rather than the properties of individual electrodes; thus, there are a small number of studies on the influence of the specifications of AgNWs and the preparation process of electrodes on the properties of AgNW transparent electrodes. Bernal studied the effect of spin speed on the optical and electrical properties of AgNW transparent electrode, but he did not study the effect of the concentration of the AgNW solution [28]. In addition, as the specifications of AgNWs and the electrode preparation processes adopted by scholars all over the world are very different, the properties of AgNW transparent electrodes in different papers vary greatly. Taking sheet resistance as an example, the initial sheet resistance of the AgNW transparent electrodes prepared by Tokuno at room temperature was 1.8 × 10^4^ Ω/sq [29], while the initial sheet resistance of the one prepared by Ha was only 87 Ω/sq [30], both without post-treatment. Their difference could already reach up to several orders of magnitude.

Due to the fact that different electronic devices have different requirements on the optical and electrical properties of the electrode, this paper adopted the spin-coating method to prepare AgNW transparent electrodes. The effects of concentration and spin speed on the uniformity, sheet resistance, transmittance, *FoM*, and *haze value* of AgNW transparent electrodes were studied, providing a reference for the rapid preparation of AgNW transparent electrodes with the required properties in the laboratory.

## 2. Materials and Methods

### 2.1. Materials

The AgNWs (MG-NW-S40) used in this experiment were purchased from Shanghai Maoguo Nanotechnology Co. LTD (Shanghai, China), and its main parameters are shown in Table 1. The substrate of the AgNW transparent electrode was 20 mm × 20 mm × 1 mm transparent quartz glass.

### 2.2. Preparation of AgNW Solutions with Different Concentrations

The AgNWs purchased were dispersed in an ethanol solution at a concentration of 5 mg/mL. The original AgNW solution was diluted with ethanol solutions in volume ratios of 1:4, 2:3, and 3:2, respectively, to obtain AgNW solutions with concentrations of 1, 2, and 3 mg/mL.

### 2.3. Preparation of AgNW Transparent Electrode

First, a 20 mm × 20 mm × 1 mm transparent quartz glass was immersed in an ethanol solution and acetone solution in turn for ultrasonic cleaning for 15 min, and then the glass was placed on a thermostatic hot plate and dried at 150 °C for 15 min. Next, the glass was placed into an ultraviolet ozone processor for 15 min, finally obtaining the clean glass substrate. Before the deposition of AgNWs on the glass substrate, the clean glass substrate was fixed on the spin coater and the AgNW solution was dispersed and homogenized using a magnetic stirrer for 1 h. During the deposition of AgNWs on the glass substrate, 100 μL of AgNW solution was taken using a pipette and dropped onto the glass substrate evenly, and the AgNW wet film was prepared by setting the spin speed and the duration. After the deposition of AgNWs on the glass substrate, and after the solvent volatilized, the AgNW transparent electrode could be obtained.

### 2.4. Characterization

A scanning electron microscope (SU8010, Hitachi, Tokyo, Japan) was used to test the morphology of the samples. An ultraviolet-visible-near-infrared spectrometer (UV3600 I Plus, Shimazu, Kyoto, Japan) was used to test the transmittance spectra and the *haze value* of the samples. The sheet resistance (Rs) of the samples was tested using a four-point probe system (Model 280, Saratoga Technology, Saratoga, NY, USA).

## 3. Results

### 3.1. Effects of Concentration and Spin Speed on the Distribution of AgNWs

The two most important photoelectric properties of transparent electrodes are electrical conductivity and transmittance. For the AgNW transparent electrode, when the specifications (length and diameter) of the AgNWs remain unchanged, its photoelectric properties are mainly affected by the distribution of AgNWs on the substrate. The AgNWs pile up and lap under the action of gravity to form a conductive network. The denser the AgNW network is, the more conductive pathways it will have and the better its conductivity will be. However, the dense AgNW network has a strong scattering effect on visible light, which is not conducive to its transmittance. As the conductivity and the transmittance of the AgNW transparent electrode are a pair of internal contradictions, it is very important to balance them by regulating the distribution of AgNWs on the substrate.

The distribution of AgNWs on the substrate is mainly affected by the concentration of the AgNW solution and the spin-coating process. The concentration of the AgNW solution determines the initial quantity density of AgNWs on the substrate, while the spin-coating process affects the spread of the AgNW solution on the substrate under centrifugal force. The uniformity of AgNWs can be changed by adjusting the spin speed and the duration. Generally, with the decrease in the concentration of the AgNW solution and the increase in the spin speed and the duration, the distribution of AgNWs on the substrate will become more dispersed and the thickness of the electrode will also decrease. Considering the influence of air turbulence on the spin process, it is generally necessary to set a sufficient duration to balance all the influencing factors. In order to ensure the uniformity of the electrode, the duration of this experiment was set at 45 s.

**Figure 1:**
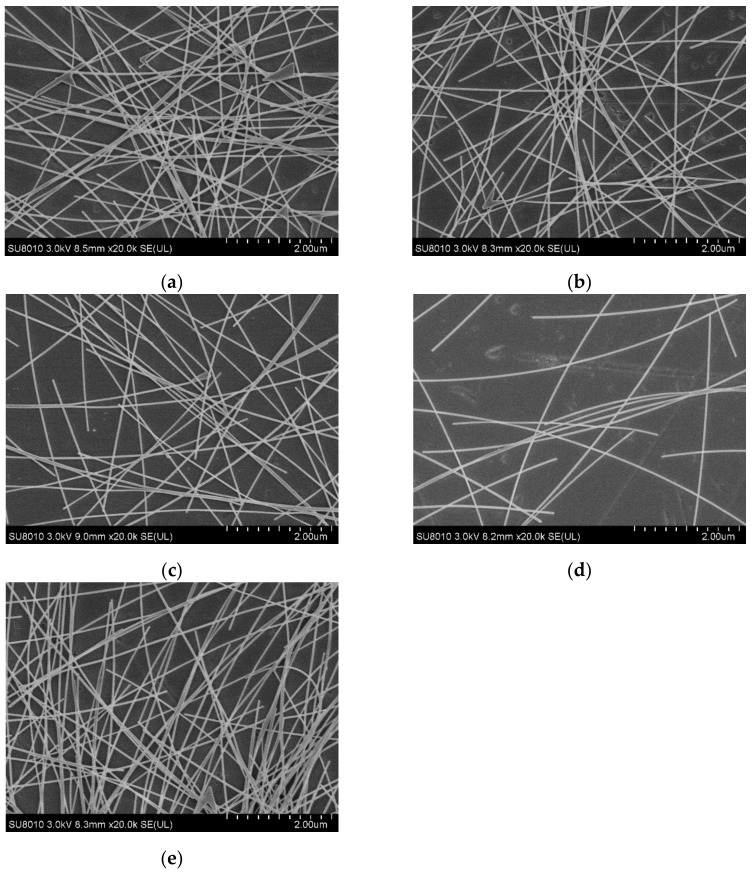
SEM morphology of AgNW transparent electrodes prepared at different concentrations and spin speeds: (**a**) 2 mg/mL and 1000 rpm; (**b**) 2 mg/mL and 2000 rpm; (**c**) 2 mg/mL and 3000 rpm; (**d**) 1 mg/mL and 2000 rpm; (**e**) 3 mg/mL and 2000 rpm, respectively.

Figure 1 shows the scanning electron microscope (SEM) morphology of AgNW transparent electrodes prepared at different concentrations and spin speeds. In Figure 1a–c, the concentration of the AgNWs solution was 2 mg/mL, and the spin speeds were 1000, 2000, and 3000 rpm, respectively, in order to study the effect of spin speed on the distribution of AgNWs. In Figure 1d,e, the spin speed was 2000 rpm and the concentrations of the AgNW solutions were 1 and 3 mg/mL, respectively, in order to study the effect of concentration on the distribution of AgNWs. As shown in Figure 1a–c, when the concentration remained unchanged (2 mg/mL), the AgNWs were densely distributed with the phenomenon of multiple wires being wound together at a relatively low spin speed (1000 rpm). With the increase in spin speed (2000 rpm), the density of AgNWs decreased slightly and the distribution of AgNWs was more uniform; meanwhile, the phenomenon of multiple wires being wound together was alleviated. The density of AgNWs on the substrate decreased significantly at a relatively higher spin speed (3000 rpm), the AgNW network was thinner, and its uniformity decreased slightly. As shown in Figure 1b,d,e, when the spin speed remained unchanged (2000 rpm), the distribution of AgNWs was very sparse at a relatively low concentration (1 mg/mL), the phenomenon of multiple wires being wound together was very rare, and there were only a few contact points between the AgNWs; as a result, there were only a small number of conductive pathways in the network. With the gradual increase in concentration (2 and 3 mg/mL), this situation gradually improved, and the density of AgNWs increased, forming more points of contact and, thus, leading to the appearance of a larger number of conductive pathways in the network.

By comparison, it can be found that the effect of concentration on the distribution of AgNWs is greater than that of the spin speed. The effect of viscosity on the film forming process should be introduced here. Generally, for the AgNW solution, the higher the concentration, the higher the viscosity, which means that the solution has a higher flow resistance, the inner radial flow of droplets on the substrate surface from inside to outside is weaker, and it is not easy for the solution to migrate to the substrate edge. In addition, as the solvent (alcohol) evaporates, the viscosity of the solution will increase further. When the AgNW solution is dropped onto the substrate with a pipette gun, the AgNWs will pile up on the substrate under the action of gravity and get close to each other by van der Waals forces. During the spin-coating process, under the action of the centrifugal force, the solution will be further spread on the substrate, and the distribution of AgNWs on the substrate will be reconstructed. After the solvent completely volatilizes, the AgNW transparent electrode will be obtained. In general, the higher the viscosity of the AgNWs solution, the thicker the AgNW electrode, and the concentration of the solution will play an important role.

In general, when the concentration of the AgNW solution was 2 mg/mL and the spin speed was set at 2000 rpm, the quantity density of AgNWs on the glass substrate was moderate and the distribution of AgNWs was uniform in all directions. There were also fewer agglomerations between AgNWs, which was a relatively ideal situation.

### 3.2. Effects of Concentration and Spin Speed on the Sheet Resistance of Electrode

Sheet resistance is an important indicator of conductivity for the transparent electrode, with units of Ω/sq, and the smaller the sheet resistance is, the stronger the ability of the electrode to transfer charge along the plane direction. Jia defined the nonuniformity factor (*NUF*) to evaluate the standard deviation of the sheet resistance of the transparent electrode [31]. The calculation formula of *NUF* is shown in Equation (1):

(1) $$NUF=\sqrt{\frac{\sum_{i=1}^{n}{\left(R_{i}-\bar{R}\right)}^{2}}{n{\bar{R}}^{2}}}$$

where *n* represents the number of measurements, *R_i_* represents the measured value of sheet resistance, and $\bar{R}$ represents the average value of all measured values. The *NUF* can reflect the uniformity of the transparent electrode, where the smaller the *NUF*, the better the uniformity of the transparent electrode.

**Figure 2:**
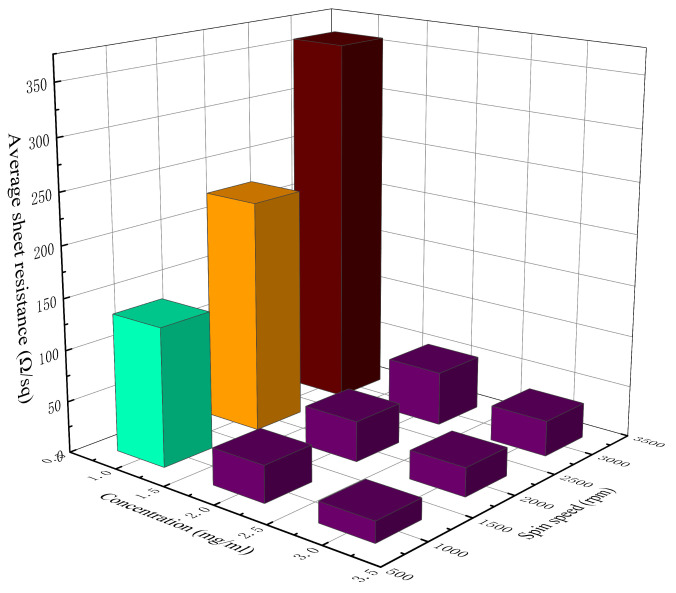
The 3D histogram of the variation in sheet resistance with concentration and spin speed.

**Figure 3:**
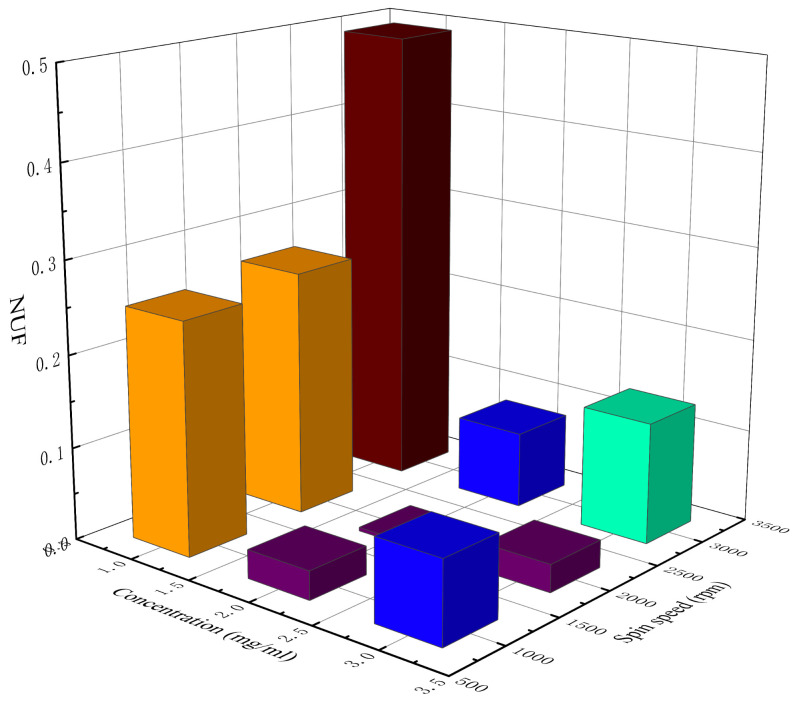
The 3D histogram of the variation in the *NUF* with concentration and spin speed.

Table 2 lists the measured sheet resistance, average sheet resistance, and the *NUF* of the AgNW transparent electrode at different concentrations and spin speeds. Figure 2 and Figure 3 show, respectively, a 3D histogram of the variation in sheet resistance and *NUF* with concentration and spin speed.

When the concentration of the AgNW solution was 1 mg/mL, the density of AgNWs on the substrate was relatively low (Figure 1d); thus, there were only a few effective conductive pathways. The sheet resistance of the electrodes at different spin speeds reached hundreds of Ω/sq, representing a relatively poor electrical conductivity. Moreover, these electrodes also had a relatively high *NUF*, meaning that they were relatively uneven and rough. At the concentration of 1 mg/mL, the sheet resistance of the AgNW transparent electrode increased rapidly with the increase in spin speed.

When the concentration of the AgNW solution was 2 mg/mL, the conductive pathways in the electrode were significantly increased (Figure 1b), and the sheet resistance of the electrode decreased significantly, by nearly an order of magnitude. As the distribution of AgNWs on the substrate became more saturated, the *NUF* of the electrode also decreased significantly, indicating that the uniformity of the AgNW transparent electrode was better at this concentration.

When the concentration of the AgNW solution was 3 mg/mL, the AgNWs were oversaturated on the substrate (Figure 1e), leading to a slight decrease in the electrode’s uniformity, and the phenomenon of multiple wires being wound together was obvious and the electrode was thicker. Although there were more conductive pathways in the network in this case, the contact resistance between the AgNWs also increased; thus, the sheet resistance of the electrode only dropped by 10–20 Ω/sq compared with the previous concentration (2 mg/mL), which was not very obvious. Meanwhile, the *NUF* of the electrode rose slightly, which indicated that the uniformity of the AgNW transparent electrode decreased slightly at a relatively high concentration.

As can be seen from Figure 2, with the increase in the concentration of the AgNW solution and the decrease in spin speed, the sheet resistance of the AgNW transparent electrode gradually decreased. As can be seen from Figure 3, when the concentration of the AgNW solution remained unchanged, the *NUFs* of the electrode at 1000 and 3000 rpm were generally larger than that at 2000 rpm, indicating that too high or too low a spin speed would affect the uniformity of the electrode. When the spin speed remained unchanged, the *NUFs* of the electrode using the AgNW solution of 1 and 3 mg/mL were generally larger than that of 2 mg/mL, indicating that too large or too small a concentration of the AgNW solution would affect the uniformity of the electrode. In general, when the concentration of the AgNW solution was 2 mg/mL and the spin speed was 2000 rpm, the *NUF* of the electrode was only 0.004, which was smallest, indicating that the uniformity of the AgNW transparent electrode was very good in this case. In addition, the electrode prepared at 1000 rpm with a concentration of 2 mg/mL and that prepared at 3000 rpm with a concentration of 3 mg/mL were very similar in terms of the average sheet resistance, which were 35.76 and 34.45 Ω/sq, respectively.

### 3.3. Effects of Concentration and Spin Speed on the Transmittance of Electrode

**Figure 4:**
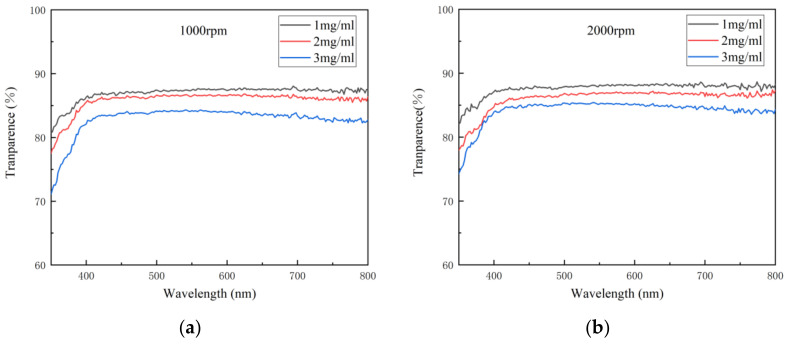

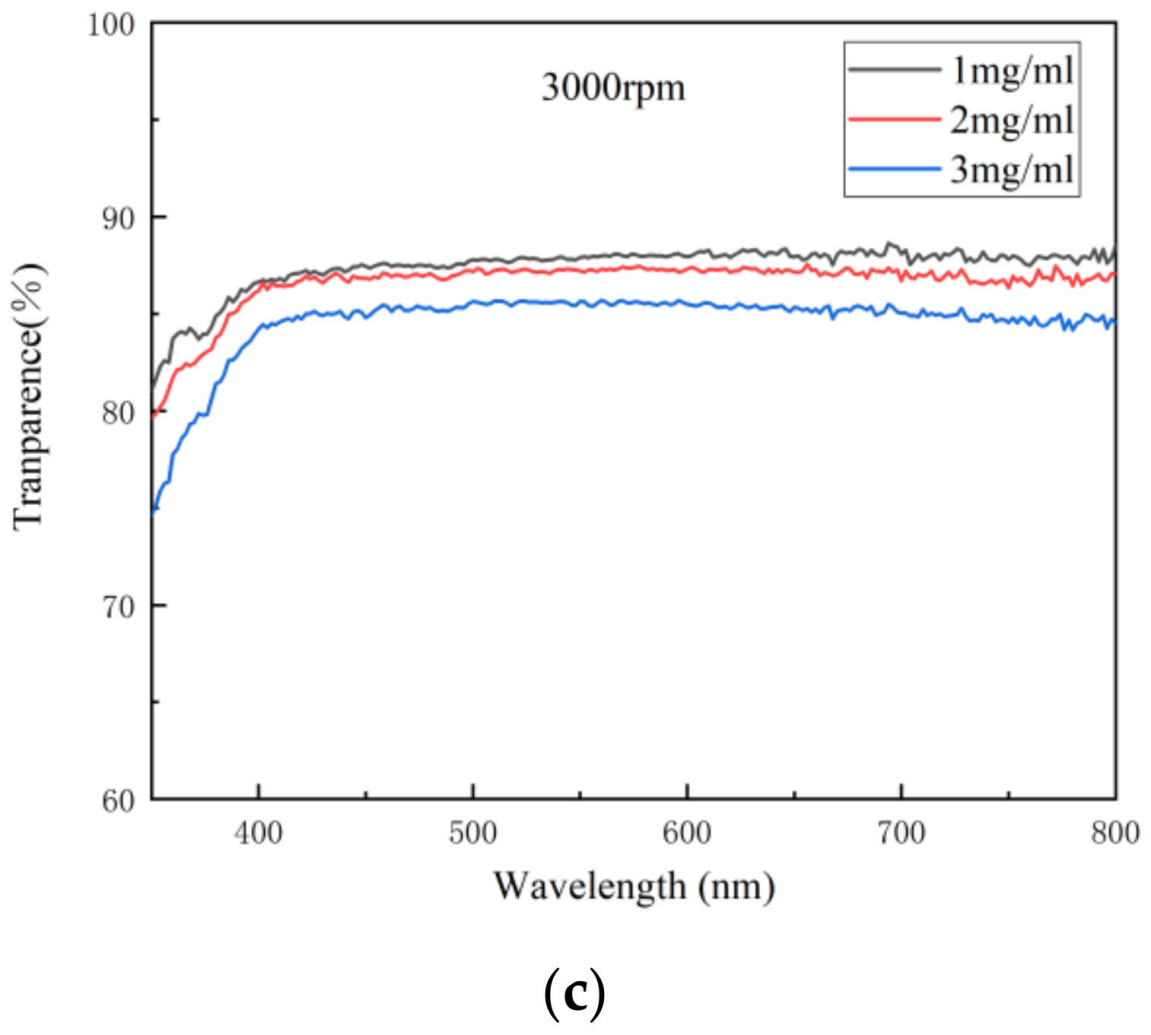
The variation in transmittance spectra with concentration at different spin speeds: (**a**) 1000; (**b**) 2000; (**c**) 3000 rpm.

Transmittance is the percentage of the luminous flux passing through the medium and the incident luminous flux. As an important index of the optical properties of the transparent electrode, transmittance indicates the ability of light of different wavelengths to pass through the electrode. When the thickness of the electrode is less than the wavelength of the incident light, only a small amount of light can be absorbed by the electrode, so in general, the thinner the electrode is, the higher its transmittance will be. Figure 4 shows the variation in transmittance spectra with concentration at different spin speeds. It can be seen from Figure 4 that the transmittance of the electrode decreased with the concentration at each spin speed, indicating that with the increase in the density of AgNWs, the ability of light of each wavelength to pass through the electrode decreased gradually.

When evaluating the transmittance of the electrode, its transmittance at 550 nm is often taken as a reference. Table 3 shows the transmittances of the AgNW transparent electrode at 550 nm at different concentrations and spin speeds. As can be seen from Table 3, the transmittance of each electrode at 550 nm was good, generally between 84.19% and 88.12%. With the increase in the concentration of the AgNW solution and the decrease in spin speed, the transmittance of the electrode at 550 nm generally decreased due to the increase in the density of the AgNW network. However, there was an exception. When the concentration of the AgNW solution was 1 mg/mL, with the increase in spin speed, the transmittance of the electrode at 550 nm first increased and then decreased, from 87.44% to 88.12% and then to 87.89%. This might be related to the uniformity of the electrode. It can be seen from above that, when the concentration of the AgNW solution was 1 mg/mL and the spin speed was 3000 rpm, the *NUF* of the electrode was very high, indicating that the electrode was very uneven and rough, where the decrease in transmittance might be caused by the nonuniformity of the electrode. In addition, the electrode prepared at 1000 rpm with a concentration of 2 mg/mL and that prepared at 3000 rpm with a concentration of 3 mg/mL were also similar in terms of transmittance, which were 86.47% and 85.63%, respectively.

### 3.4. Effects of Concentration and Spin Speed on the FoM of Electrode

Due to the inherent contradiction between the conductivity and transmittance of the AgNW transparent electrode, in order to better reflect the photoelectric characteristics of the electrode, the quality factor *FoM* is generally introduced. *FoM* is defined as the ratio between the conductivity of direct current *σ_DC_* and the photoinduced conductivity *σ_OP_*, namely *σ_DC_*/*σ_OP_*. Generally, the larger the value of *FoM*, the better the photoelectric performance of the electrode [32]. The *FoM* of an electrode with a high comprehensive quality is generally greater than 35. The relationship between transmittance, sheet resistance, and *FoM* can be expressed by Equation (2) [33]:

(2) $$T={\left[1+\frac{Z_{0}}{2R_{S}}\frac{\sigma_{OP}}{\sigma_{DC}}\right]}^{-2}$$

where *T* is the transmittance of the electrode at 550 nm, *Z*_0_ is the impedance of free space, whose value is 337 Ω, and *R_s_* is the sheet resistance of the electrode. After simplification, Equation (2) can be simplified to Equation (3):

(3) $$FoM=\frac{188.5\sqrt{T}}{\left(1-\sqrt{T}\right)R_{S}}$$

**Figure 5:**
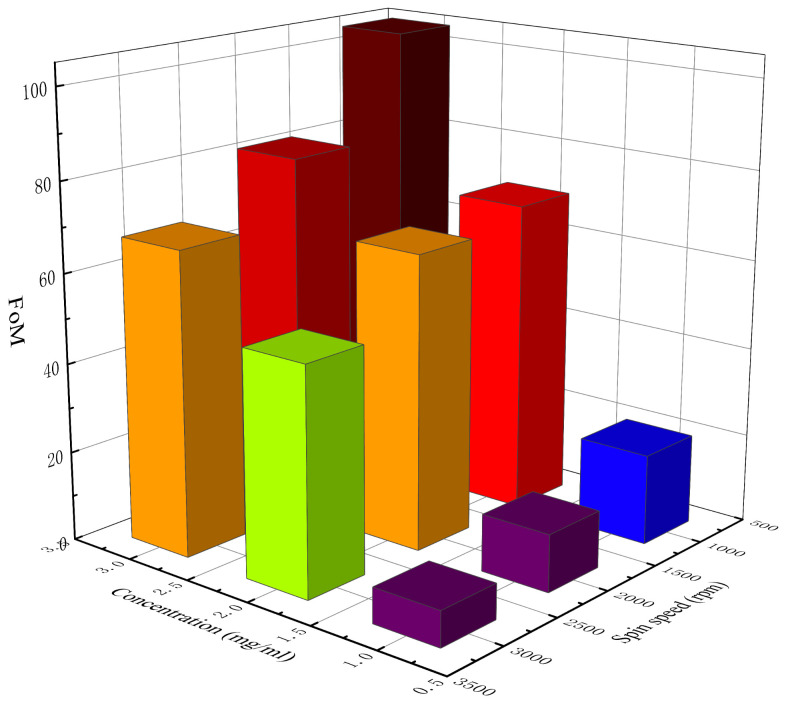
The 3D histogram of the variation in *FoM* with concentration and spin speed.

By substituting the sheet resistance (*R_s_*) and the transmittance at 550 nm (*T*) into Equation (3), the *FoM* of each electrode can be calculated. Table 4 lists the calculated *FoM* of the AgNW transparent electrodes at different concentrations and spin speeds, and Figure 5 shows the 3D histogram of the variation in *FoM* with concentration and spin speed. According to Table 4 and Figure 5, the *FoM* of different electrodes varied greatly. When the concentration of AgNW solution was 1 mg/mL, the *FoM* of each electrode was all less than 35 at different spin speeds, indicating that this concentration was not suitable for the preparation of the AgNW transparent electrode with high comprehensive properties. When the concentrations of the AgNW solution were 2 and 3 mg/mL, the *FoM* of each electrode was all more than 35 at different spin speeds, indicating that for the AgNWs with a diameter of 40 nm and a length of 45 μm (aspect ratio: 1125), the concentration of the AgNW solution should reach at least 2 mg/mL to make an electrode with a relatively high comprehensive quality. Among all the prepared electrodes, when the concentration of the AgNW solution was 3 mg/mL and the spin speed was 1000 rpm, the *FoM* of the electrode was highest, reaching 104.42, showing great potential. The results showed that the *FoM* of the electrode generally decreased with the spin speed, indicating that the spin speed should not be too fast. As the concentration of the AgNW solution increased, the *FoM* of the electrode generally increased, indicating that when the concentration of the AgNW solution was low, with the increase in the concentration, the positive impact of the improvement of conductivity on the *FoM* was greater than the negative impact of the decrease in transmittance on the *FoM*. As the concentration continues to increase, there will theoretically be a threshold that makes the *FoM* reach its maximum value, which will not be studied further in this paper. In addition, the electrode prepared at 1000 rpm with a concentration of 2 mg/mL and that prepared at 3000 rpm with a concentration of 3 mg/mL were also similar in terms of the *FoM*, which were 69.92 and 67.84, respectively.

### 3.5. Effects of Concentration and Spin Speed on the Haze Value of Electrode

The *haze value* is the percentage of the light flux of diffusion and the light flux passing through the medium. As another important index of the optical performance of transparent electrodes, the *haze value* indicates the ability of the diffuse light to pass through the electrode. The *haze value* can be calculated by Equation (4) [34]:

(4) $$Haze=\left(T_{tot}-T_{spec}\right)/T_{tot}\times 100\%$$

where *T_tot_* is the total transmittance of light and *T_spec_* is the specular transmittance of light. A high *haze value* indicates a high degree of light scattering, which may lead to a cloudy appearance of the electrode. Due to the disordered distribution of nanowires on the substrate, it is easy for nanowire-based transparent electrodes to generate light diffusion, so their *haze values* are generally high.

**Figure 6:**
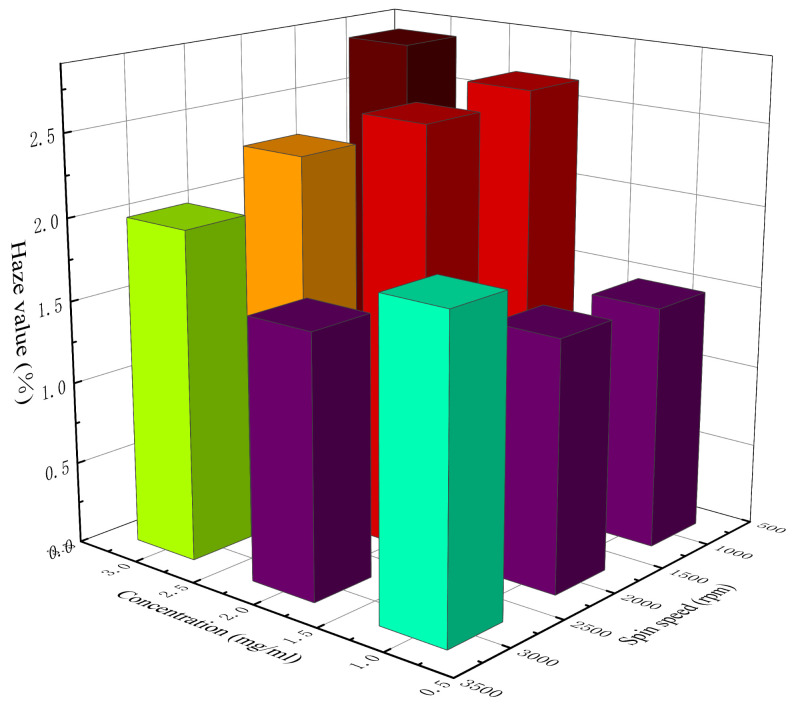
The 3D histogram of the variation in *haze value* with concentration and spin speed.

Table 5 lists the calculated *haze value* of AgNW transparent electrodes at different concentrations and spin speeds. Figure 6 shows a 3D histogram of the variation in *haze value* with concentration and spin speed. It can be seen from Table 5 and Figure 6 that the clarity of each electrode was good and the *haze value* was generally low, with the lowest being 1.48% and the highest being 2.82%. When the concentration of the AgNW solution was 1 mg/mL, the *haze value* presented an increasing trend with the increase in spin speed, which might be related to the uniformity of the distribution of AgNWs on the substrate. As can be seen above, at a relatively low concentration, with the increase in spin speed, the uniformity of electrode gradually decreased, and then the rough surface of the electrode enhanced the scattering of light, which led to the increase in *haze value*. When the concentrations of the AgNW solution were 2 and 3 mg/mL, the *haze value* presented a decreasing trend with the increase in spin speed, which might be related to the density of AgNWs on the substrate. As can be seen above, at a relatively higher concentration, the distribution of AgNWs on the substrate was generally uniform. With the increase in spin speed, the density of AgNWs on the substrate and the thickness of the electrode gradually decreased; thus, the scattering effect of the AgNW network on light was weakened, which led to a decrease in *haze value*. In addition, the electrode prepared at 1000 rpm with a concentration of 2 mg/mL and that prepared at 3000 rpm with a concentration of 3 mg/mL were also similar in terms of the *haze value*, which were 2.65% and 2%, respectively. The results showed that although the concentration and the spin speed of these two electrodes were different during the preparation, their photoelectric properties were very similar; thus, they could be considered equivalent.

## 4. Conclusions

(1) The distribution of AgNWs on the substrate increased in density with the increase in the concentration of the AgNW solution and the decrease in spin speed. The effect of concentration on the distribution of AgNWs was greater than that of spin speed. Under different preparation conditions, the transmittance of each electrode was generally between 84.19% and 88.12% at 550 nm, the average sheet resistance was between 20.09 and 358.11 Ω/sq, the highest *FoM* was 104.42, and the lowest *haze value* was 1.48%.
(2) Among the prepared electrodes, when the concentration of the AgNW solution was 2 mg/mL and the spin speed was 2000 rpm, the electrode was the most uniform, and its test performance was the most stable. When the concentration of the AgNW solution was 1 mg/mL, the *haze value* of the electrode was positively correlated with the spin speed; when the concentrations of the AgNW solution were 2 and 3 mg/mL, the *haze value* of the electrode was negatively correlated with the spin speed.
(3) The electrode prepared at 1000 rpm with a concentration of 2 mg/mL and that prepared at 3000 rpm with a concentration of 3 mg/mL were very similar in terms of the average sheet resistance, transmittance at 550 nm, *FoM*, and *haze value*; thus, these two electrodes could be considered equivalent.
(4) When the concentration of the AgNW solution was low, with the increase in the concentration, the positive impact of the improvement of conductivity on the *FoM* was greater than the negative impact of the decrease in transmittance on the *FoM*. For the AgNWs used in this experiment with a diameter of 40 nm and a length of 45 μm (aspect ratio: 1125), the concentration of the AgNW solution should reach at least 2 mg/mL to ensure that the *FoM* of the electrode is greater than 35.

## Figures and Tables

**Table 1:** Main parameters of silver nanowires (AgNWs).

Specification	Diameter nm	Length μm	Aspect Ratio	Purity %	Appearance	Solvent	Concentration
MG-NW-S40	40	45	1125	99.9	Incanus	Ethanol	5 mg/mL

**Table 2:** Measured sheet resistance, average sheet resistance, and the nonuniformity factor (*NUF*) of AgNW transparent electrode at different concentrations and spin speeds.

Concentration mg/mL	Spin Speed rpm	Measured Sheet Resistance (1) Ω/sq	Measured Sheet Resistance (2) Ω/sq	Measured Sheet Resistance (3) Ω/sq	Average Sheet Resistance Ω/sq	*NUF*
1 mg/mL	1000	104.25	117.52	181.55	134.44	0.251
	2000	264.69	140.85	269.77	225.10	0.265
	3000	523.48	436.7	114.16	358.11	0.492
2 mg/mL	1000	37.01	34.34	35.93	35.76	0.031
	2000	39.26	39.46	39.11	39.28	0.004
	3000	46.58	55.4	56.04	52.67	0.082
3 mg/mL	1000	17.82	22.2	20.26	20.09	0.089
	2000	28.37	26.62	28.39	27.79	0.030
	3000	40.78	31.98	30.6	34.45	0.131

**Table 3:** The transmittance of the AgNW transparent electrode at 550 nm at different concentrations and spin speeds.

Concentration mg/mL	Spin Speed rpm	Transmittance
1 mg/mL	1000	87.44%
	2000	88.12%
	3000	87.89%
2 mg/mL	1000	86.47%
	2000	86.90%
	3000	87.21%
3 mg/mL	1000	84.19%
	2000	85.19%
	3000	85.63%

**Table 4:** The *FoM* of the AgNW transparent electrode at different concentrations and spin speeds.

Concentration mg/mL	Spin Speed rpm	*FoM*
1 mg/mL	1000	20.20
	2000	12.83
	3000	7.90
2 mg/mL	1000	69.92
	2000	65.98
	3000	50.53
3 mg/mL	1000	104.42
	2000	81.29
	3000	67.84

**Table 5:** The *haze value* of the AgNW transparent electrode at different concentrations and spin speeds.

Concentration mg/mL	Spin Speed rpm	*Haze Value*
1 mg/mL	1000	1.48%
	2000	1.52%
	3000	1.90%
2 mg/mL	1000	2.65%
	2000	2.59%
	3000	1.59%
3 mg/mL	1000	2.82%
	2000	2.27%
	3000	2%

## Data Availability

The data presented in this study are available on request from the corresponding author.
